# Prognostic significance of programmed cell death‐ligand 1 expression on circulating tumor cells in various cancers: A systematic review and meta‐analysis

**DOI:** 10.1002/cam4.4236

**Published:** 2021-08-23

**Authors:** Yushu Ouyang, Wendao Liu, Ningning Zhang, Xiaobing Yang, Jinwei Li, Shunqin Long

**Affiliations:** ^1^ Department of Intervention The Second Clinical Medical College of Guangzhou University of Chinese Medicine Guangdong Provincial Hospital of Chinese Medicine Guangzhou China; ^2^ Department of Oncology Heping Hospital Affiliated to Changzhi Medical College Changzhi China; ^3^ Department of Oncology The Second Clinical Medical College of Guangzhou University of Chinese Medicine Guangdong Provincial Hospital of Chinese Medicine Guangzhou China

**Keywords:** circulating tumor cells, immune checkpoint inhibitors, overall survival, programmed cell death‐ligand 1, progression‐free survival

## Abstract

**Background:**

The prognostic significance of programmed cell death‐ligand 1 (PD‐L1) expression on circulating tumor cells (CTCs) has been explored but is still in controversy. We performed, for the first time, a meta‐analysis to systematically evaluate its prognostic value in human cancers.

**Methods:**

Literature databases were searched for eligible studies prior to June 30, 2021. The pooled hazard ratios (HRs) and 95% confidence intervals (95% CIs) were calculated for the associations of pre‐treatment and post‐treatment PD‐L1^+^ CTCs with progression‐free survival (PFS) and overall survival (OS). Subgroup analyses with regards to cancer type, treatment, CTC enrichment method, PD‐L1 detection method, cut‐off, and specifically the comparison model were performed.

**Results:**

We included 30 eligible studies (32 cohorts, 1419 cancer patients) in our analysis. Pre‐treatment PD‐L1^+^ CTCs detected by immunofluorescence (IF) tended to predict better PFS (HR = 0.55, 95% CI 0.28–1.08, *p* = 0.084) and OS (HR = 0.61, 95% CI 0.36–1.04, *p* = 0.067) for immune checkpoint inhibitor (ICI) treatment, but were significantly associated with unfavorable survival for non‐ICI therapies (PFS: HR = 1.85, 95% CI 1.21–2.85, *p* = 0.005; OS: HR = 2.44, 95% CI 1.69–3.51, *p* < 0.001). Post‐treatment PD‐L1^+^ CTCs predicted markedly worse PFS and OS. The prognostic value was obviously modulated by comparison models. Among patients with detectable CTCs, PD‐L1^+^ individuals had comparable survival to PD‐L1^−^ individuals, except ICI treatment for which PD‐L1^+^ may predict better PFS (HR = 0.42, 95% CI 0.17–1.06, *p* = 0.067). Patients with PD‐L1^+^ CTCs had worse survival prognosis compared to those without PD‐L1^+^ CTCs in overall analysis (PFS: HR = 2.10, 95% CI 1.59–2.77, *p* < 0.001; OS: HR = 2.55, 95% CI 1.70–3.81, *p* < 0.001) and in most subgroups.

**Conclusions:**

Our analysis demonstrated that PD‐L1 positive expression on CTCs predicted better survival prognosis for ICI treatment but worse survival for other therapies, which thus can be potentially used as a prognostic marker of malignant tumor treatment. However, the prognostic value of PD‐L1^+^ CTCs for ICI treatment needs validation by more large‐scale studies in the future.

## Graphical abstract

Meta‐analysis shows that positive expression of PD‐L1 on circulating tumor cells predicts better survival prognosis in cancer patients treated with immune checkpoint inhibitors but unfavorable survival in those treated with other therapies. PD‐L1 expression on CTCs may be a promising prognostic marker for tumor treatment.

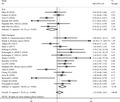

## 1 INTRODUCTION

Programmed cell death‐ligand 1 (PD‐L1) overexpression on tumor tissues has been explored as a promising biomarker that predicts response to immune checkpoint inhibitors (ICIs) therapy.^1^ Patients with PD‐L1 overexpression may benefit more from anti‐ PD‐1/PD‐L1 antibodies.^2^ Immunohistochemistry (IHC)‐based tests for PD‐L1 expression on tumor tissues can help select patients suitable for these drugs.^3^ However, the predictive role of tumor PD‐L1 expression is still in controversy and some limitations need to be overcome. About 10% of patients negative for PD‐L1 tumor expression can also benefit from ICIs therapy,^4^ and the underlying mechanism needs further investigation. There is obvious spatial and temporal heterogeneity of PD‐L1 expression on tumor tissues. The expression may significantly vary from tumor boundary to core, differ between primary and metastatic sites,^5^, ^6^ and dynamically change along with disease progression.^7^ Therefore, the biopsy at a single tumor site or a certain time point may not be sufficiently representative of the overall PD‐L1 status of tumor tissue. Since tumor tissue biopsy is invasive and may increase the risk of tumor metastasis, multi‐site or longitudinal biopsies of tumor tissue, however, are considered not applicable.^8^

In view of the shortages of PD‐L1 expression detected by conventional tissue biopsy and IHC, researchers have recently focused on circulating PD‐L1 expressions in serum, plasma, circulating tumor cells (CTCs), and exosomes. These alternative methods allow a minimally invasive and real‐time detection for a more accurate representation of the heterogenous expression of PD‐L1, and are feasible for dynamic monitoring of PD‐L1 status during anti‐cancer treatment.^9^ A recent meta‐analysis involving 21 studies demonstrated that higher soluble PD‐L1 (sPD‐L1) was significantly correlated with worse survivals in various cancers.^10^ Significantly higher levels of serum/plasma‐derived exosomal PD‐L1 were found in melanoma, non‐small cell lung cancer (NSCLC), head, and neck squamous cell carcinoma (HNSCC) than in healthy controls.^11^, ^12^, ^13^ Patients with elevated exosomal PD‐L1 level were less likely to respond to immunotherapy^12^ and had shorter survivals^14^, ^15^ than those with low levels. Thus, sPD‐L1 and exosomal PD‐L1 may be potential biomarkers for cancer therapies.^16^

PD‐L1 expression on CTCs was first demonstrated in breast cancer,^17^ and then reported in colorectal cancer, bladder cancer, NSCLC, HNSCC, and melanoma.^18^, ^19^, ^20^, ^21^, ^22^ Subsequently, the clinical significance of PD‐L1^+^ CTCs was explored. Wang Y et al found that PD‐L1^+^ CTCs were associated with significantly shorter progression‐free survival (PFS) in NSCLC patients undergoing radiochemotherapy.^23^ Liu MY et al showed that gastric cancer patients with higher number of PD‐L1^+^ CTCs had decreased PFS and worse overall survival (OS) than those with lower number of PD‐L1^+^ CTCs.^24^ Winograd P et al demonstrated that PD‐L1^+^ CTCs predicted inferior OS in hepatocellular carcinoma patients undergoing ICIs therapy.^25^ However, some researches have yielded inconsistent and even opposite results. Tada H et al observed prolonged survival in HNSCC patients with PD‐L1^+^ CTCs.^26^ In colorectal cancer patients receiving regorafenib, a receptor tyrosine kinases inhibitor, PD‐L1^+^ CTCs predicted favorable survivals.^27^ These results indicated a controversial prognostic value of PD‐L1 expression on CTCs in human cancers.

The inconsistent results may be caused by many factors, such as the difference in cancer type, anti‐cancer treatment, CTC enrichment method, or PD‐L1 detection method. Here, we performed the first meta‐analysis to systematically assess the prognostic role of PD‐L1 expression on CTCs in various cancers.

## 2 MATERIALS AND METHODS

### 2.1 Studies selection

This study is performed in accordance with the Preferred Reporting Items for Systematic Reviews and Meta‐analysis (PRISMA).^28^ We comprehensively searched PubMed, Web of Science, and EMBASE prior to June 30, 2021, using the following search items: (CTCs OR neoplastic circulating cells OR CTCs) AND (programmed death ligand 1 OR PD‐L1 OR CD274). Studies investigating the association between PD‐L1^+^ CTCs and survival in cancer patients were a candidate for the present meta‐analysis. The references of relevant articles were manually reviewed for additional candidate studies.

Candidate articles meeting the following criteria were included in the meta‐analysis: (1) enriched and isolated CTCs in blood samples of cancer patients and detected PD‐L1 expression on CTCs; (2) reported hazard ratio (HR) and 95% confidence interval (95% CI) of PD‐L1^+^ CTCs in association with PFS and/or OS, or provided sufficient data to calculate HR and 95% CI. Reviews, meta‐analyses, case reports, and duplicated studies were excluded. Since this is a meta‐analysis, ethical approval is not required.

### 2.2 Data extraction

Two independent researchers extracted the following information of eligible studies: first author, publication year, cancer type, anti‐cancer therapy, enrichment method and platform of CTCs, detection method and antibody of PD‐L1, metastatic status, prognostic cut‐off of PD‐L1^+^ CTCs, cell‐surface vimentin (CSV) expression on CTCs, time point of blood draw, HR, and 95% CI of survival outcomes. Discrepancies, if occurred, were resolved by discussion.

### 2.3 Quality assessment

Newcastle‐Ottawa Scale was used to assess the quality of eligible studies in three categories: selection, comparability, and outcome. A total of nine stars were distributed to the assessment items and six or more stars indicated a high quality.

### 2.4 PD‐L1 expression status on CTCs and comparison model

As some cancer patients had undetectable CTCs, the PD‐L1 expression status can be divided into three categories: CTCs negative (status 1), CTC positive plus PD‐L1 negative expression on CTCs (status 2: PD‐L1^−^ CTCs), CTC positive plus PD‐L1 positive expression on CTCs (status 3: PD‐L1^+^ CTCs). Therefore, the prognostic value of PD‐L1^+^ CTCs, that is, status 3, can be analyzed under two comparison models. The first comparison model was performed among CTCs positive patients, that is, those with status 3 and those with status 2 (model 1: CTC PD‐L1^+^ vs. CTC PD‐L1^−^). The second comparison model was performed in all patients, that is, those with status 3 and those with status 1+2 (model 2: presence of PD‐L1^+^ CTCs vs. absence of PD‐L1^+^ CTCs).

### 2.5 Statistical analysis

We assessed the heterogeneity by *I* ^2^ and *Q* test. *I* ^2^ <50% with *p* value of *Q* test >0.10 indicated no obvious heterogeneity, and then a fixed‐effect model was applied to combine HR and 95% CI of survival outcomes. Otherwise, a random‐effect model was used. Subgroups analyses regarding prognostic cut‐off of PD‐L1^+^ CTCs, cancer type, CTC enrichment method, metastatic status, treatment, comparison model, CSV expression status, PD‐L1 detection method were performed. Moreover, we analyzed the interactions between comparison models and the other variables. Sensitivity analysis was performed, and funnel plot and Egger's test were used to assess publication bias. The present meta‐analysis was performed by STATA 12.0 (StataCorp).

## 3 RESULTS

### 3.1 Baseline features of eligible studies

**FIGURE 1:**
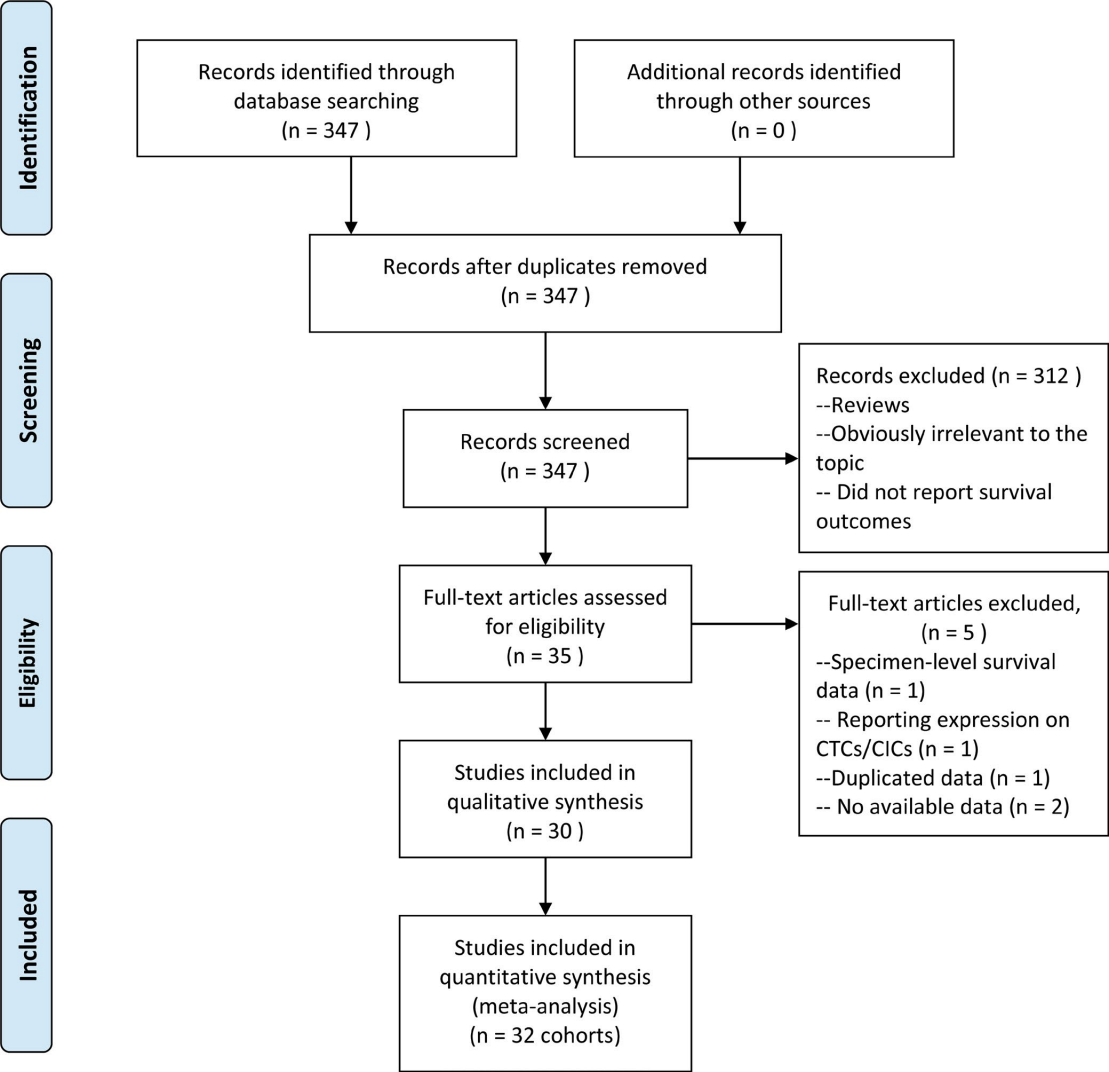
Flow chart for the literature search and study selection. CIC, circulating immune cell; CTC, circulating tumor cell

Three hundred and fourteen articles were identified through literature search, and 35 studies were remained after discarding studies not relevant to the research topic. Furtherly, we excluded five candidate studies for the following reasons: one only provided specimen‐level survival data,^29^ one reported the correlation of PD‐L1^+^ CTCs/circulating immune cells with survival,^30^ one was duplicated with another study,^18^, ^31^ and two did not report survival outcomes.^32^, ^33^ Finally, we identified 30 studies eligible for the present meta‐analysis^18^, ^19^, ^20^, ^21^, ^22^, ^23^, ^24^, ^25^, ^26^, ^27^, ^34^, ^35^, ^36^, ^37^, ^38^, ^39^, ^40^, ^41^, ^42^, ^43^, ^44^, ^45^, ^46^, ^47^, ^48^, ^49^, ^50^, ^51^, ^52^, ^53^ as shown in Figure 1. A total of 1419 patients with malignant tumors, including 208 breast cancer, 253 gastrointestinal cancer, 194 genitourinary cancer, 161 head and neck cancer, 25 melanoma, 12 metastatic neuroendocrine tumor, and 566 NSCLC, were analyzed. CTCs were enriched by epithelial cell adhesion molecule (EpCAM)‐based or size‐based methods or were enrichment‐free in 11, 11, and 8 studies, respectively. PD‐L1 was detected for protein expression on CTCs by immunofluorescence (IF) in 25 studies, and for mRNA expression in 5 studies. All studies detected pre‐treatment PD‐L1^+^ CTCs, while five studies also detected post‐treatment PD‐L1^+^ CTCs. The cut‐off of ≥1 PD‐L1^+^ CTCs was the most commonly used by 14 studies to define PD‐L1 positive patients. As to the comparison model, 15 studies used model 1 while 15 studies used model 2. Specifically, two researches^22^, ^46^ both had two cohorts of patients with different cancers, then each cohort was included as an individual study into quantitative analysis. The characteristics of all eligible studies are summarized in Table 1.

**TABLE 1:** Baseline characteristics of all studies included in meta‐analysis

Study	Cancer	Therapy	CTC enrichment	PD‐L1 detection (antibody)	PD‐L1^+^ CTC patient		Time point of blood draw, outcome	NOS
					Cut‐off	Number		
Satelli A (2016)	Metastatic colon cancer, prostate cancer	Chemotherapy	Enrichment‐free	IF (AHP‐1703, AbD Serotec)	≥50% PD‐L1^+^ CTCs	41/61 (67.2%)^a^, 23/30 (76.7%)^a^	Pre‐treatment, PFS and OS	6
Anantharaman A (2016)	Metastatic bladder cancer	Chemotherapy, ICI and others	Enrichment‐free	IF (E1L3N, Cell Signaling)	>1 PD‐L1^+^ CTCs/ml	4/19 (21.0%)^b^	Pre‐treatment, OS	6
Boffa DJ (2017)	Stage I–IV NSCLC	NR	Enrichment‐free	IF (E1L3N, Cell Signaling)	>1.1 PD‐L1^+^ CTCs/ml	14/112 (12.5%)^a^	Pre‐treatment, OS	7
Adams DL (2017)	Stage I–IV NSCLC	Radiotherapy	Size‐based (CellSieve)	IF (130021, R&D system)	≥2 API	15/34 (44.2%)^b^	Pre‐ and post‐treatment, PFS	8
Strati A (2017)	Locally advanced HNSCC	Chemoradiotherapy	EpCAM‐based (CellSearch)	RT‐qPCR	Relative fold change	24/94 (25.5%)^a^	Pre‐ and post‐treatment, PFS and OS	7
Kallergi G (2018)	Metastatic NSCLC	Chemotherapy	Size‐based (ISET)	IF (B7‐H1, Novus Biologicals)	>3 PD‐L1^+^ CTCs/ml	2/30 (6.7%)^a^	Pre‐treatment, PFS	7
Dhar M (2018)	Metastatic NSCLC	Pembrolizumab, nivolumab, avelumab	Size‐based (Vortex HT chip)	IF (4059, ProSci Inc)	≥2 PD‐L1^+^ CTCs	7/17 (41.2%)^a^	Pre‐treatment, PFS	6
Guibert N (2018)	Metastatic NSCLC	Nivolumab	Size‐based (ISET)	IF (D8T4X, Cell Signaling)	≥1% PD‐L1^+^ CTCs	74/89 (83.1%)^b^	Pre‐treatment, PFS and OS	8
Yue CY (2018)	Advanced gastrointestinal tumors	Sintilimab	EpCAM‐based (Pep@MNPs)	IF (KN802, Kohnoor)	≥20% PD‐L1^+^ CTCs	14/35 (40.0%)^b^	Pre‐ and post‐treatment, PFS	8
Kulasinghe A (2018)	Stage I–IV HNSCC, metastatic NSCLC	Chemotherapy, ICI, TKIs	Size‐based (ClearCell)	IF (28‐–2, Abcam)	≥1 PD‐L1^+^ CTCs	6/11 (54.5%)^b^, 11/17 (64.7%)^b^	Pre‐treatment, PFS	7
Wang Y (2019)	Non‐metastatic NSCLC	Radiotherapy, chemoradiotherapy	EpCAM‐based (GO chip)	IF (329802, BioLegend)	≥5% PD‐L1^+^ CTCs	6/13 (46.2%)^b^	Pre‐treatment, PFS	7
Manjunath Y (2019)	Stage I–IIIA NSCLC	Surgery	Size‐based (CellSieve)	IF (D8T4X, Cell Signaling)	≥3 PD‐L1^+^ CTCs	18/30 (60.0%)^a^	Pre‐treatment, OS	6
Kotsakis A (2019)	Metastatic NSCLC	Chemotherapy	Size‐based (ISET)	IF (BioLegend)	≥1 PD‐L1^+^ CTCs	7/34 (20.6%)^a^	Pre‐treatment, PFS	8
Dong JS (2019)	Stage I–III NSCLC	Surgery	Size‐based (CanPatrol)	RNA‐ISH	≥1 PD‐L1^+^ CTCs	56/110 (50.1%)^b^	Pre‐treatment, OS	6
Liu MY (2020)	Advanced gastric cancer	Chemotherapy	EpCAM‐based (Miltenyi Biotec)	IF (Cell Signaling)	≥8 PD‐L1^+^ CTCs/ml	18/32 (56.2%)^a^	Pre‐treatment, PFS and OS	7
Papadaki MA (2020)	Metastatic breast cancer	Chemotherapy, hormone therapy	Enrichment‐free	IF (E1L3N, Cell Signaling)	≥1 P PD‐L1^+^CTCs	5/98 (5.1%)^a^	Pre‐treatment, PFS and OS	7
Tada H (2020)	Stage I–IV HNSCC	NR	Size‐based (CellSieve)	RT‐qPCR	2^−ΔΔ^ *^Ct^* >1	11/28 (39.3%)^b^	Pre‐treatment, PFS	8
Pinato DJ (2020)	Neuroendocrine tumor	Surgery	EpCAM‐based (CellSearch)	IF (FAB1561P, R&D System)	≥1 PD‐L1^+^ CTCs	9/12 (75.0%)^a^	Pre‐treatment, OS	8
Khattak MA (2020)	Metastatic melanoma	Pembrolizumab	Enrichment‐free	IF	≥1 PD‐L1^+^ CTCs	16/25 (60.0%)^b^	Pre‐treatment, PFS and OS	7
Cheng YX (2020)	Stage II–IV NSCLC	Initial treated	Size‐based (ISET)	IF (28‐8, Abcam)	≥1% PD‐L1^+^ CTCs	22/41 (53.6%)^b^	Pre‐treatment, PFS	8
Bergmann S (2020)	Advanced urothelial carcinoma	NR	EpCAM‐based (CellSearch)	IF (E1L3N, Cell Signaling)	≥1 PD‐L1^+^ CTCs	4/16 (25.0%)^b^	Pre‐treatment, OS	7
Papadaki MA (2020)	Metastatic NSCLC	ICI	Size‐based (Parsortix)	IF (E1L3N, Cell Signaling)	≥1 PD‐L1^+^ CTCs	3/15 (20.0%)^a^	Pre‐treatment, PFS and OS	6
Jacot W (2020)	Metastatic breast cancer	NR	EpCAM‐based (CellSearch)	IF (FAB1561P, R&D System)	≥1 PD‐L1^+^ CTCs	26/72 (36.1%)^a^	Pre‐treatment, PFS and OS	6
Raimondi L (2020)	Metastatic colorectal cancer	Regorafenib	EpCAM‐based (CellSearch)	IF (D8T4X, Cell Signaling)	≥1 PD‐L1^+^ CTCs	24/38 (63.2%)^b^	Pre‐treatment, PFS	7
Winograd P (2020)	Hepatocellular carcinoma	NR	EpCAM‐based (NanoVelcro chip)	IF (R&D System)	≥1 PD‐L1^+^ CTCs	31/87 (35.6%)^a^	Pre‐treatment, OS	8
Chalfin HJ (2020)	Metastatic genitourinary cancer	Cabozantinib, nivolumab, ipilimumab	Enrichment‐free	IF (E1L3N, Cell Signaling)	≥1 PD‐L1^+^ CTCs	7/67 (10.4%)^a^	Pre‐treatment, PFS Post‐treatment, OS	8
Tada H (2020)	Recurrent/metastatic HNSCC	Nivolumab	Enrichment‐free	RT‐qPCR	40^−Δ^ *^Ct^* >24.98	16/28 (57.1%)^b^	Pre‐treatment, OS	6
Polioudaki H (2020)	Metastatic breast caner	Eribulin	Enrichment‐free	IF (E1L3N, Cell Signaling)	≥1 PD‐L1^+^ CTCs	5/38 (13.2%)^a^	Pre‐ and post‐treatment, PFS and OS	7
Zavridou M (2021)	mCRPC	Chemotherapy, new hormonal agents	EpCAM‐based (Dynabeads Epithelial Enrich)	RT‐qPCR	Relative fold change	34/62 (54.8%)^b^	Pre‐treatment, OS	7
Dall'Olio FG (2021)	Advanced NSCLC	nivolumab, pembrolizumab, atezolizumab	EpCAM‐based (CellSearch)	IF (MIH3, BioLegend)	≥1 PD‐L1^+^ CTCs	13/24 (54.2%)^b^	Pre‐treatment, PFS and OS	7

Abbreviations: API: average pixel intensity of immunofluorescence staining; CTC: circulating tumor cell; HNSCC: head and neck squamous cell carcinoma; ICI: immune checkpoint inhibitor; IF: immunofluorescence; mCRPC: metastatic castration‐resistant prostate cancer; NOS: Newcastle‐Ottawa Scale; NR: not reported;NSCLC: non‐small cell lung cancer; OS: overall survival; PD‐L1: programmed cell death ligand 1; PFS: progression‐free survival; RNA‐ISH, RNA in situ hybridization; RT‐qPCR: real‐time quantitative polymerase chain reaction.

^a^ Percentage of patients with PD‐L1^+^ CTCs in all patients.

^b^ Percentage of patients with PD‐L1^+^ CTCs in CTC positive patients.

### 3.2 Correlation between pre‐treatment PD‐L1^+^ CTCs and survival of cancer patients

**FIGURE 2:**
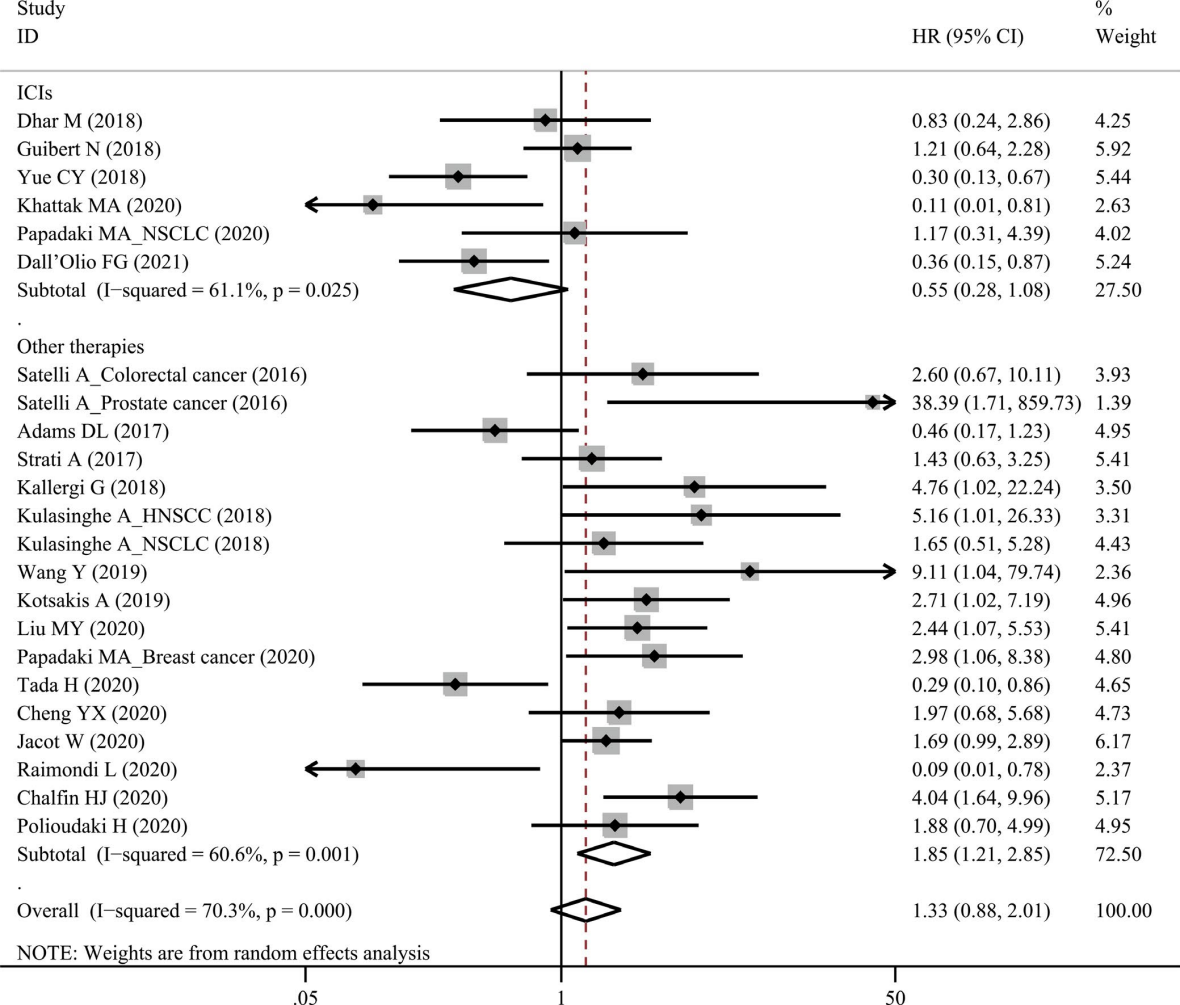
Forest plot of pre‐treatment PD‐L1^+^ circulating tumor cells with progression‐free survival. ICI, immune checkpoint inhibitor; PD‐L1, programmed cell death‐ligand 1

Twenty‐three studies comprising 992 patients evaluated the association of pre‐treatment PD‐L1^+^ CTCs with PFS (Table 2). There was obvious heterogeneity (*I* ^2^ = 70.3%, *p* < 0.001) and a random‐effect model was applied. Pre‐treatment PD‐L1^+^ CTCs were not associated with PFS (HR = 1.33, 95% CI 0.88–2.01, *p* = 0.170). When stratified for treatment, we found that PD‐L1^+^ CTCs detected by IF were associated with a better PFS (HR = 0.55, 95% CI 0.28–1.08, *p* = 0.084, Figure 2) for ICI treatment in a borderline significance, but a worse PFS for other therapies (HR = 1.85, 95% CI 1.21–2.85, *p* < 0.001, Figure 2).

**TABLE 2:** Association between pre‐treatment PD‐L1^+^ CTCs and progression‐free survival in cancers

Pre‐treatment, PFS	No. of studies	No. of patients	Combined HR (95% CI)	*p*	Heterogeneity		Model
					*I* ^2^ (%)	*p*	
Overall	23	992	1.33 (0.88–2.01)	0.170	70.3	<0.001	RE
Treatment							
ICIs	6	210	0.55 (0.28–1.08)	0.084	61.1	0.025	RE
Other therapies	17	782	**1.85 (1.21–2.85)**	**0.005**	60.6	<0.001	RE
Cancer type							
NSCLC	10	319	1.30 (0.76–2.21)	0.341	58.0	0.011	RE
Breast cancer	3	208	**1.90 (1.24–2.91)**	**0.003**	0	0.635	FE
Gastrointestinal cancer	4	210	0.74 (0.17–3.14)	0.684	84.6	<0.001	RE
Genitourinary cancer	2	97	**4.81 (2.02–11.45)**	**<0.001**	46.2	0.173	FE
HNSCC	3	133	1.18 (0.28–2.09)	0.826	79.2	0.008	RE
Enrichment method							
EpCAM‐based	7	346	0.92 (0.41–208)	0.847	80.9	<0.001	RE
Size‐based	10	321	1.30 (0.77–2.20)	0.326	56.1	0.015	RE
Enrichment‐free	6	325	2.25 (0.92–5.52)	0.077	64.1	0.016	RE
Metastatic disease							
Yes	14	642	**1.70 (1.09–2.64)**	**0.019**	54.5	0.008	RE
Mixed	9	350	1.00 (0.49–2.06)	0.991	77.1	<0.001	RE
Comparison							
CTC PD‐L1^+^ versus CTC PD‐L1^−^	11	355	0.71 (0.37–1.37)	0.307	72.4	<0.001	RE
Presence versus absence of PD‐L1^+^ CTCs	12	637	**2.10 (1.59–2.77)**	**<0.001**	7.0	0.377	FE
Vimentin expression							
Yes	4	205	**2.47 (1.41–4.33)**	**0.002**	9.1	0.347	FE
Not specified	19	787	1.15 (0.73–1.82)	0.542	72.3	<0.001	RE
Prognostic cut‐off							
≥1 PD‐L1^+^ CTCs	12	480	1.43 (0.83–2.46)	0.202	67.5	<0.001	RE
Other cut‐offs	11	512	1.27 (0.68–2.38)	0.458	72.7	<0.001	RE
PD‐L1 detection							
IF	21	870	1.44 (0.93–2.22)	0.101	69.9	<0.001	RE
mRNA expression	2	122	0.67 (0.14–3.20)	0.616	81.1	0.021	RE

Statistically significant values are indicated in bold.

Abbreviations: CSV, cell‐surface vimentin; FE, fixed‐effect model; HNSCC, head and neck squamous cell carcinoma; HR, hazard ratio; ICIs, immune checkpoint inhibitors; IF, Immunofluorescence;NSCLC, non‐small cell lung cancer; PFS, progression‐free survival; RE, random‐effect model.

**FIGURE 3:**
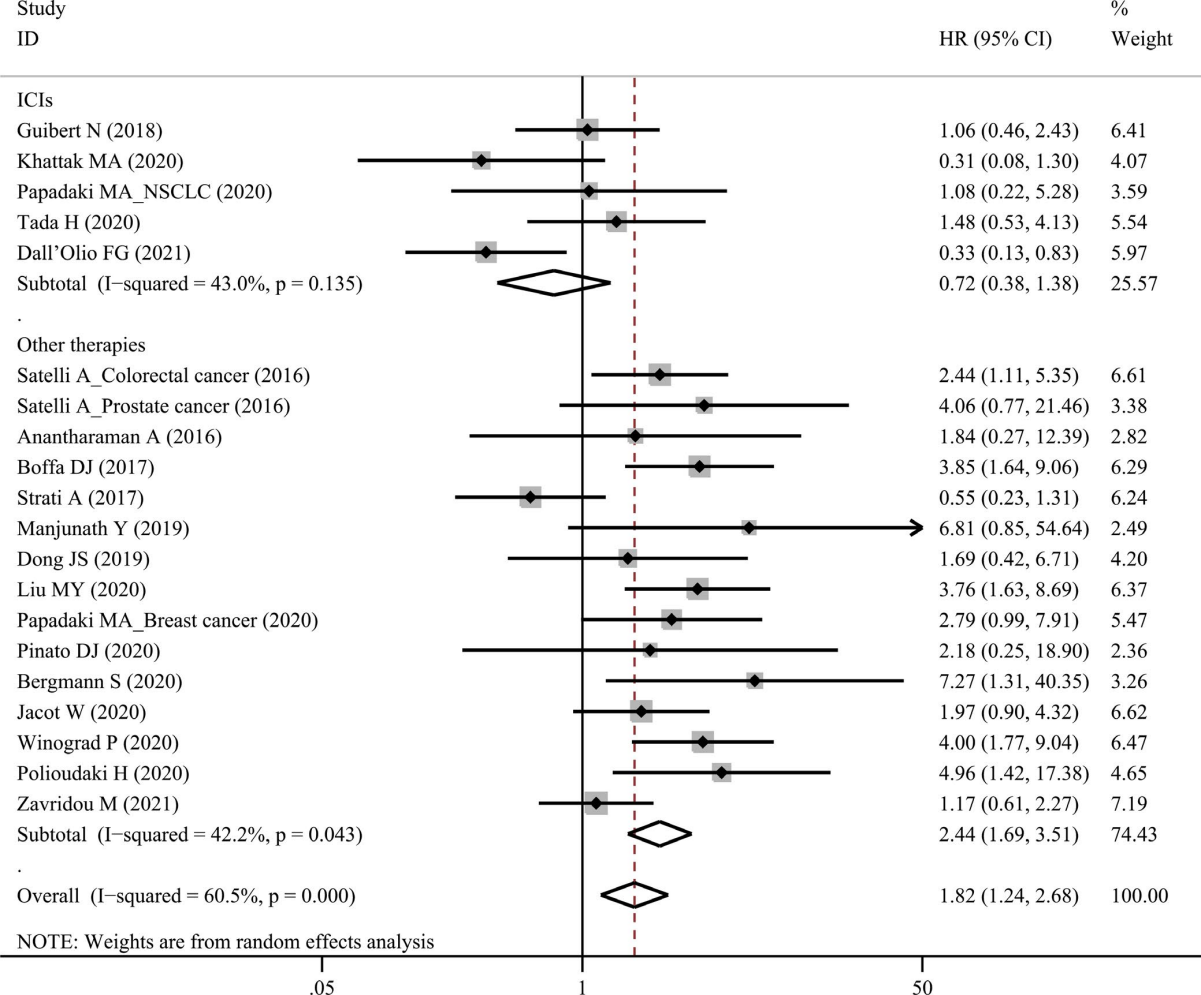
Forest plot of pre‐treatment PD‐L1^+^ circulating tumor cells with overall survival. ICI, immune checkpoint inhibitor; PD‐L1, programmed cell death‐ligand 1

The association between pre‐treatment PD‐L1^+^ CTCs and OS was evaluated in 20 studies comprising 1096 patients (Table 3). Pooled analysis using a random‐effect model demonstrated that patients with PD‐L1^+^ CTCs had significantly worse OS (HR1.82, 95% CI 1.24–2.68, *p* = 0.002). When stratified for treatment, PD‐L1^+^ CTCs seemed to predict a better OS (HR = 0.72, 95% CI 0.38–1.38, *p* = 0.325, Figure 3) for ICI treatment, but were significantly associated with worse OS for other therapies (HR = 2.44, 95% CI 1.69–3.51, *p* < 0.001, Figure 3). If we excluded only one study^35^ detecting PD‐L1 mRNA and remained the other studies detecting PD‐L1 by IF, we found that PD‐L1^+^ CTCs had a borderline association with prolonged OS (HR = 0.61, 95% CI 0.36–1.04, *p* = 0.067) for ICI treatment.

**TABLE 3:** Association between pre‐treatment PD‐L1^+^ CTCs and overall survival in cancers

Pre‐treatment, OS	No. of studies	No. of patients	Combined HR (95% CI)	*p*	Heterogeneity		Model
					*I* ^2^ (%)	*p*	
Overall	20	1096	**1.82 (1.24–2.68)**	**0.002**	60.5	<0.001	RE
Treatment							
ICIs	5	181	0.72 (0.38–1.38)	0.325	43.0	0.135	RE
Other therapies	15	915	**2.44 (1.69–3.51)**	**<0.001**	42.2	0.043	RE
Cancer type							
NSCLC	6	380	1.43 (0.59–3.46)	0.424	71.3	0.004	RE
Breast cancer	3	208	**2.62 (1.50–4.59)**	**0.001**	0	0.467	FE
Gastrointestinal cancer	3	224	**3.29 (2.06–5.26)**	**<0.001**	0	0.645	FE
Genitourinary cancer	4	125	1.69 (0.97–2.93)	0.063	40.5	0.169	FE
HNSCC	2	122	0.87 (0.33–2.28)	0.773	52.1	0.148	RE
Enrichment method							
EpCAM‐based	8	435	1.64 (0.82–3.28)	0.166	76.6	<0.001	RE
Size‐based	4	244	1.38 (0.74–2.56)	0.312	0	0.421	FE
Enrichment‐free	8	417	**2.37 (1.61–3.50)**	**<0.001**	40.3	0.110	FE
Metastatic disease							
Yes	12	555	**1.65 (1.22–2.22)**	**0.001**	21.3	0.234	FE
Mixed	8	541	2.12 (0.93–4.81)	0.074	79.3	<0.001	RE
Comparison							
CTC PD‐L1^+^ versus CTC PD‐L1^−^	8	371	1.06 (0.60–1.89)	0.840	53.2	0.037	RE
Presence versus absence of PD‐L1^+^ CTCs	12	725	**2.55 (1.70–3.81)**	**<0.001**	41.4	0.065	RE
CSV expression							
Yes	5	235	**3.46 (2.13–5.61)**	**<0.001**	0	0.816	FE
Not specified	15	861	1.45 (0.93–2.27)	0.099	62.0	0.001	RE
Prognostic cut‐off							
≥1 PD‐L1^+^ CTCs	11	584	1.65 (0.91–3.00)	0.101	66.4	0.001	RE
Other cut‐offs	9	512	**2.02 (1.21–3.37)**	**0.007**	55.5	0.022	RE
PD‐L1 detection							
IF	16	802	**2.13 (1.36–3.35)**	**0.001**	60.3	0.001	RE
mRNA expression	4	294	1.04 (0.67–1.62)	0.852	4.2	0.372	FE

Statistically significant values are indicated in bold.

Abbreviations: CSV, cell‐surface vimentin; FE, fixed‐effect model; HNSCC, head and neck squamous cell carcinoma; HR, hazard ratio; ICI, immune checkpoint inhibitor; IF, Immunofluorescence; NSCLC, non‐small cell lung cancer; PFS, progression‐free survival; RE, random‐effect model.

### 3.3 Subgroup analyses of pre‐treatment PD‐L1^+^ CTCs in association with survival

We performed subgroup analysis according to the cancer type (NSCLC, breast cancer, gastrointestinal cancer, genitourinary cancer, HNSCC), CTC enrichment method (EpCAM‐based, size‐based, enrichment‐free), metastatic status (yes, mixed), comparison model (model 1 and model 2), CSV expression (yes, no specified), prognostic cut‐off (≥1 PD‐L1^+^ CTCs, other cut‐offs), and PD‐L1 detection method (IF, mRNA expression). The results of subgroup analyses for PFS and OS are shown in Tables 2 and 3, respectively.

#### 3.3.1 Cancer type

**FIGURE 4:**
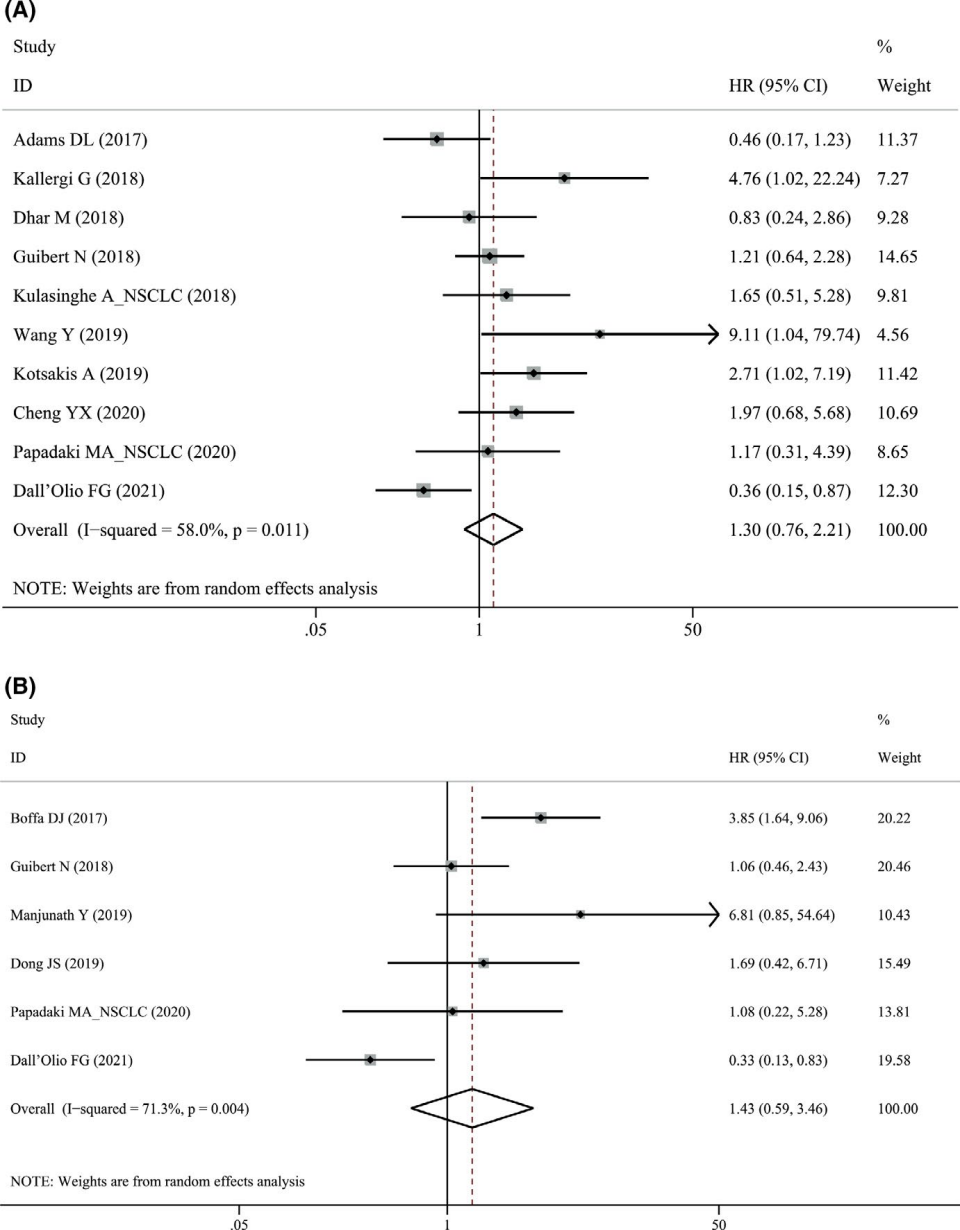
Forest plots of pre‐treatment PD‐L1^+^ circulating tumor cells with (A) progression‐free survival and (B) overall survival in patients with non‐small cell lung cancer. PD‐L1, programmed cell death‐ligand 1

In NSCLC, PD‐L1^+^ CTCs were neither associated with PFS (HR = 1.30, 95% CI 0.76–2.21, *p* = 0341, Figure 4A) nor OS (HR = 1.43, 95% CI 0.59–3.46, *p* = 0.424, Figure 4B). We further stratified the analysis for treatment and found that ICIs‐treated patients with PD‐L1^+^ CTCs seemed to have prolonged survival (PFS: HR = 0.84, 95% CI 0.54–1.31, *p* = 0.442; OS: HR = 0.68, 95% CI 0.38–1.20, *p* = 0.184) although it did not reach statistical significance. In contrast, in NSCLC patients treated by other therapies, PD‐L1^+^ CTCs predicted worse survival (PFS: HR = 1.96, 95% CI 0.91–4.22, *p* = 0.086; OS: HR = 3.34, 95% CI 1.68–6.64, *p* = 0.001).

Pre‐treatment PD‐L1^+^ CTCs were associated with inferior PFS in breast cancer (HR = 1.90, 95% CI 1.24–2.91) and genitourinary cancer (HR = 4.81, 95% CI 2.02–11.45), predicted significantly worse OS in breast (HR = 2.62, 95% CI 1.50–4.59), and gastrointestinal cancer (HR = 3.29, 95% CI 2.06–5.26), respectively. No association was found between PD‐L1^+^ CTCs and survival in HNSCC.

#### 3.3.2 CTC enrichment method

PD‐L1^+^ CTCs were not associated with PFS in any subgroup of the enrichment method. However, PD‐L1^+^ CTCs predicted worse OS in enrichment‐free studies (HR = 2.37, 95% CI 1.61–3.50).

#### 3.3.3 Metastatic disease, CSV expression, and treatment

**FIGURE 5:**
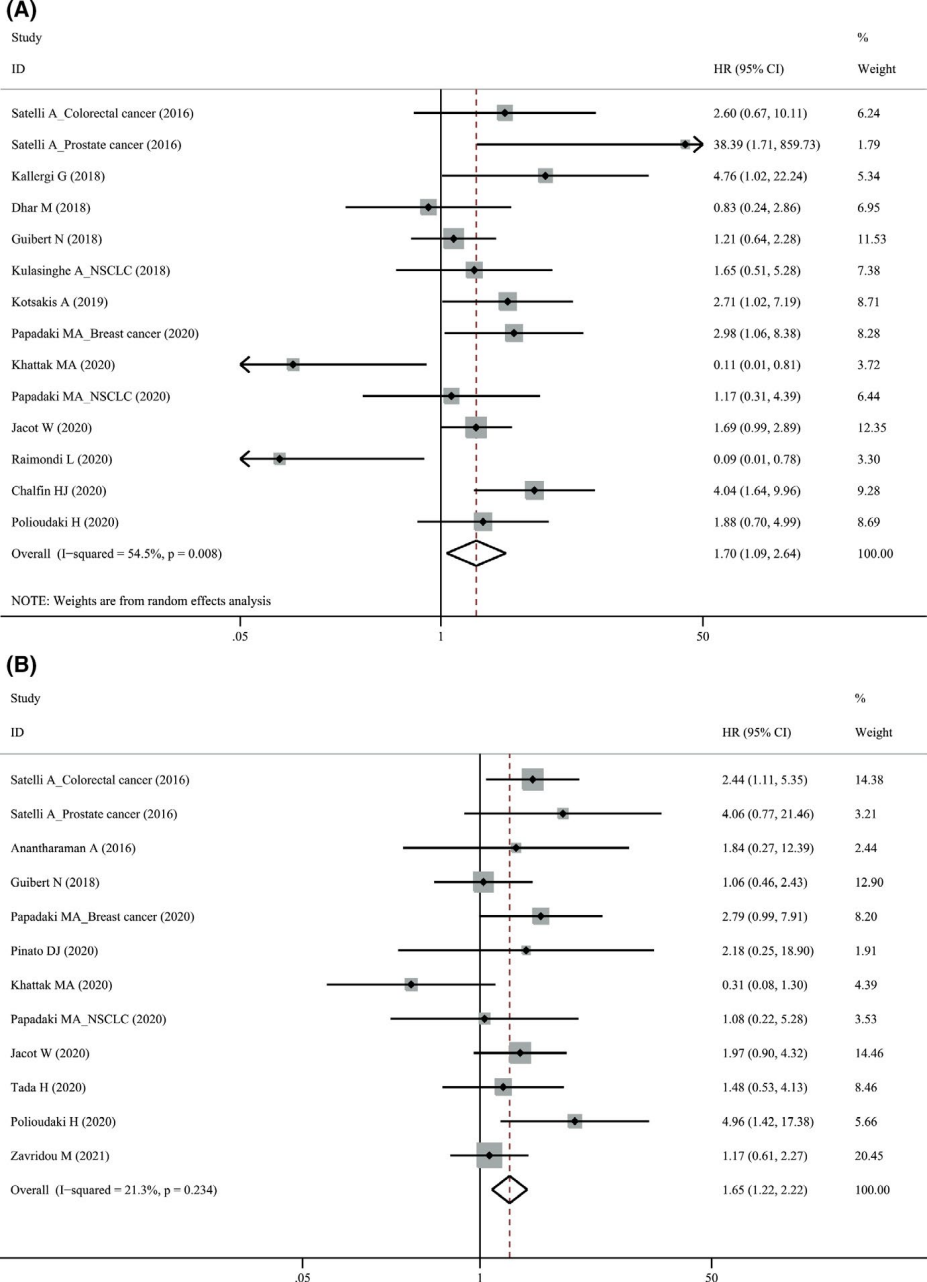
Forest plots of pre‐treatment PD‐L1^+^ circulating tumor cells with (A) progression‐free survival and (B) overall survival in patients with metastatic tumors. PD‐L1, programmed cell death‐ligand 1

Meta‐analysis of studies enrolling patients with metastatic tumors revealed significant associations of PD‐L1^+^ CTCs with worse PFS (HR = 1.70, 95% CI 1.09–2.64) and OS (HR = 1.65, 95% CI 1.22–2.22) as shown in Figure 5. Previous studies found that CTCs undergoing epithelial‐to‐mesenchymal transition (EMT) were associated with invasion and metastasis and had increased expression of mesenchymal markers such as CSV.^54^ Subgroup analysis involving five cohorts of patients^22^, ^24^, ^34^, ^45^ showed that patients with CSV^+^ PD‐L1^+^ CTCs had markedly worse PFS (HR = 2.47, 95% CI 1.41–4.33) and OS (HR = 3.46, 95% CI 2.13–5.61).

#### 3.3.4 Prognostic cut‐off and detection method of PD‐L1

Using ≥1 PD‐L1^+^ CTCs as cutoff, PD‐L1^+^ CTCs were not associated with PFS (HR = 1.43, 95% CI 0.83–2.46) or OS (HR = 1.65, 95% CI 0.91–3.00) by random‐effect model. Using the other cutoffs, PD‐L1^+^ CTCs only predicted an unfavorable OS (HR = 2.02, 95% CI 1.21–3.37). Most of the included studies detected PD‐L1 by IF, which showed worse OS (HR = 2.13, 95% CI 1.36–3.35) in patients with PD‐L1^+^ CTCs by meta‐analysis.

#### 3.3.5 Comparison model

**FIGURE 6:**
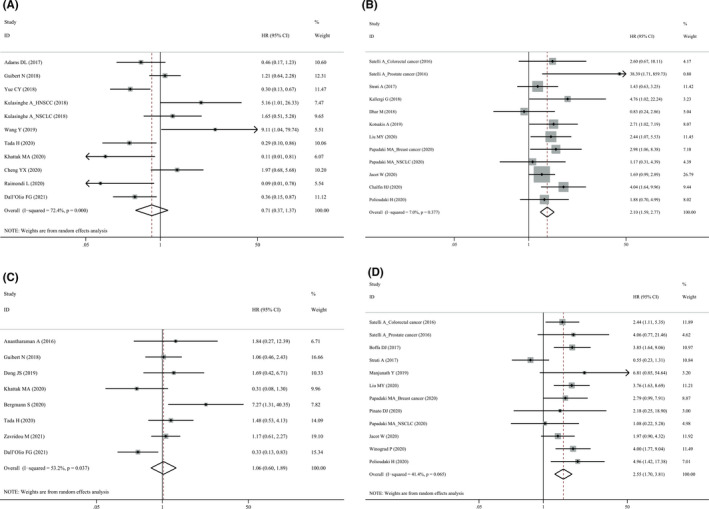
Forest plots of pre‐treatment PD‐L1^+^ CTCs with (A) progression‐free survival (PFS) under comparison model 1, (B) PFS under comparison model 2, (C) overall survival (OS) under comparison model 1 and (D) OS under comparison model 2. Comparison model 1: PD‐L1^+^ versus PD‐L1^−^ among patients with detectable CTCs. Comparison model 2: Presence versus absence of PD‐L1^+^ CTCs. CTC, circulating tumor cell; PD‐L1, programmed cell death‐ligand 1

Under model 1, there was no survival difference between CTC PD‐L1^+^ and CTC PD‐L1^−^ patients (Figure 6A,C). Under comparison model 2, patients with PD‐L1^+^ CTCs had unfavorable PFS (HR = 2.10, 95% CI 1.59–2.77, *p* < 0.001, Figure 6B) and OS (HR = 2.55, 95% CI 1.70–3.81, *p* < 0.001, Figure 6D) than those without PD‐L1^+^ CTCs. Moreover, after excluding only one study detecting PD‐L1 mRNA expression,^19^ there was no between‐study heterogeneity, and the association of PD‐L1^+^ CTCs with OS was still statistically significant (HR = 3.05, 95% CI 2.23–4.16, *p* < 0.001, *I* ^2^ = 0).

The results may indicate diverse predictive roles of PD‐L1^+^ CTCs under different comparison models and a potential source of heterogeneity from the models. Therefore, we performed further subgroup analyses under each model to investigate the interactions between the models and other variables (Table 4). Under model 1, there was huge heterogeneity in most of the subgroups, and the correlations between CTC PD‐L1^+^ and survival were not significant. Interestingly, among patients with detectable CTCs and who received ICIs, PD‐L1 positive expression had borderline association with prolonged PFS compared to negative expression (HR = 0.42, 95% CI 0.17–1.06, *p* = 0.067). In contrast, there was very low between‐study heterogeneity and PD‐L1^+^ CTCs were associated with significantly inferior survival in most of the subgroups under model 2.

**TABLE 4:** Interactions between comparison models and the other variables

Comparison model	Other variables	No. of studies and patients	*I* ^2^	HR (95% CI)	*p*
CTC PD‐L1^+^ versus CTC PD‐L1^−^	Cutoff: ≥1 PD‐L1^+^ CTCs				
	PFS	6 (156)	75.2	0.72 (0.24–2.20)	0.568
	OS	5 (262)	69.5	0.95 (0.36–2.48)	0.912
	Cutoff: other cutoffs				
	PFS	5 (199)	74.2	0.66 (0.28–1.57)	0.349
	OS	3 (109)	0	1.29 (0.76–2.20)	0.345
	Metastatic disease: yes				
	PFS	4 (169)	71.4	0.53 (0.15–1.82)	0.311
	OS	5 (223)	0	1.08 (0.70–1.65)	0.727
	Metastatic disease: mixed				
	PFS	7 (186)	74.7	0.82 (0.35–1.89)	0.635
	OS	3 (148)	81.8	1.42 (0.24–8.45)	0.697
	PD‐L1 detection: IF				
	PFS	10 (327)	73.1	0.79 (0.39–1.58)	0.501
	OS	5 (171)	68.5	0.94 (0.34–2.55)	0.897
	Treatment: ICIs				
	PFS	4 (173)	74.0	0.42 (0.17–1.06)	0.067
	OS	4 (166)	55.6	0.67 (0.31–1.45)	0.314
	Treatment: other therapies				
	PFS	7 (182)	73.0	1.05 (0.40–2.79)	0.922
	OS	4 (205)	22.3	1.54 (0.90–2.64)	0.117
	NSCLC				
	PFS	6 (218)	64.7	1.07 (0.53–2.16)	0.850
	OS	3 (233)	60.0	0.78 (0.31–1.98)	0.604
	CTC enrichment: EpCAM‐based				
	PFS	4 (110)	64.0	0.46 (0.14–1.54)	0.209
	OS	3 (100)	81.5	1.19 (0.30–4.64)	0.805
	CTC enrichment: size‐based				
	PFS	6 (220)	64.0	1.08 (0.53–2.17)	0.839
	OS	2 (199)	0	1.20 (0.59–2.44)	0.617
	CTC enrichment‐free				
	OS	3 (72)	43.1	0.98 (0.46–2.10)	0.960
Presence versus absence of PD‐L1^+^ CTCs	Cutoff: ≥1 PD‐L1^+^ CTCs				
	PFS	6 (324)	0	**2.15 (1.52–3.04)**	**<0.001**
	OS	6 (322)	0	**2.74 (1.78–4.24)**	**<0.001**
	Cutoff: other cutoffs				
	PFS	6 (313)	34.6	**2.01 (1.27–3.19)**	**0.003**
	OS	6 (403)	66.0	**2.51 (1.21–5.20)**	**0.013**
	Metastatic disease: yes				
	PFS	10 (473)	16.7	**2.17 (1.58–2.98)**	**<0.001**
	OS	7 (332)	0	**2.47 (1.63–3.74)**	**<0.001**
	Metastatic disease: mixed				
	PFS	2 (164)	0	**1.87 (1.05–3.33)**	**0.035**
	OS	4 (393)	74.7	**2.67 (1.13–6.33)**	**0.026**
	PD‐L1 detection: IF				
	PFS	11 (543)	8.1	**2.21 (1.64–2.96)**	**<0.001**
	OS	11 (631)	0	**3.05 (2.23–4.16)**	**<0.001**
	Treatment: ICIs				
	PFS	2 (37)	0	0.97 (0.39–2.40)	0.954
	OS	1 (15)	‐	1.08 (0.22–5.25)	0.924
	Treatment: other therapies				
	PFS	10 (600)	0	**2.27 (1.70–3.04)**	**<0.001**
	OS	11 (710)	43.4	**2.66 (1.76–4.04)**	**<0.001**
	NSCLC				
	PFS	4 (101)	27.0	**1.86 (1.01–3.42)**	**0.046**
	OS	3 (157)	19.5	**3.19 (1.57–6.48)**	**0.001**
	CTC enrichment: EpCAM‐based				
	PFS	3 (236)	0	**1.77 (1.19–2.62)**	**0.004**
	OS	5 (335)	70.3	2.04 (0.93–4.47)	0.075
	CTC enrichment: size‐based				
	PFS	4 (101)	27.0	**1.86 (1.01–3.42)**	**0.046**
	OS	2 (45)	47.4	2.12 (0.60–7.50)	0.242
	CTC enrichment‐free				
	PFS	5 (300)	0	**3.04 (1.83–5.06)**	**<0.001**
	OS	5 (345)	0	**3.23 (2.06–5.08)**	**<0.001**

Statistically significant values are indicated in bold.

Abbreviations: CTC, circulating tumor cell; HR, hazard ratio;ICIs, immune checkpoint inhibitors; IF, immunofluorescence; NSCLC, non‐small cell lung cancer; OS, overall survival; PD‐L1, programmed cell death ligand 1; PFS, progression‐free survival.

Previous studies have demonstrated that CTCs were independent prognostic factors for cancer treatment.^55^, ^56^ Thus, we asked whether the prognostic role of PD‐L1^+^ CTCs under comparison model 2 was largely dependent on the predictive role of CTCs. If so, there should be a correlation between the effect size, that is, HR, of both markers. We included seven studies that reported the associations of both markers with PFS^22^, ^24^, ^34^, ^38^, ^42^, ^49^ and OS.^22^, ^24^, ^34^, ^37^, ^38^, ^42^ Meta‐regression analysis showed that HRs for CTCs did not modify the effect sizes of PD‐L1^+^ CTCs with PFS and OS (*p* = 0.870 and 0.410, respectively). Furthermore, we compared the pooled effect sizes of both markers with survival outcomes. Meta‐analysis of PFS yielded HR of 1.74 (95% CI 1.23–2.47, *p* = 0.002, *I* ^2^ = 19.3%) for CTCs and 2.06 (95% CI 1.34–3.18, *p* = 0.001, *I* ^2^ = 14.0%) for PD‐L1^+^ CTCs. Meta‐analysis of OS yielded HR of 1.82 (95% CI 1.31–2.51, *p* < 0.001, *I* ^2^ = 0) for CTCs and 2.70 (95% CI 1.85–3.94, *p* < 0.001, *I* ^2^ = 0) for PD‐L1^+^ CTCs. The effect sizes for PD‐L1^+^ CTCs were slightly larger than those for CTCs. These results indicated an independent prognostic role of PD‐L1^+^ CTCs under comparison model 2.

### 3.4 Correlation between post‐treatment PD‐L1^+^ CTCs and survival

**FIGURE 7:**
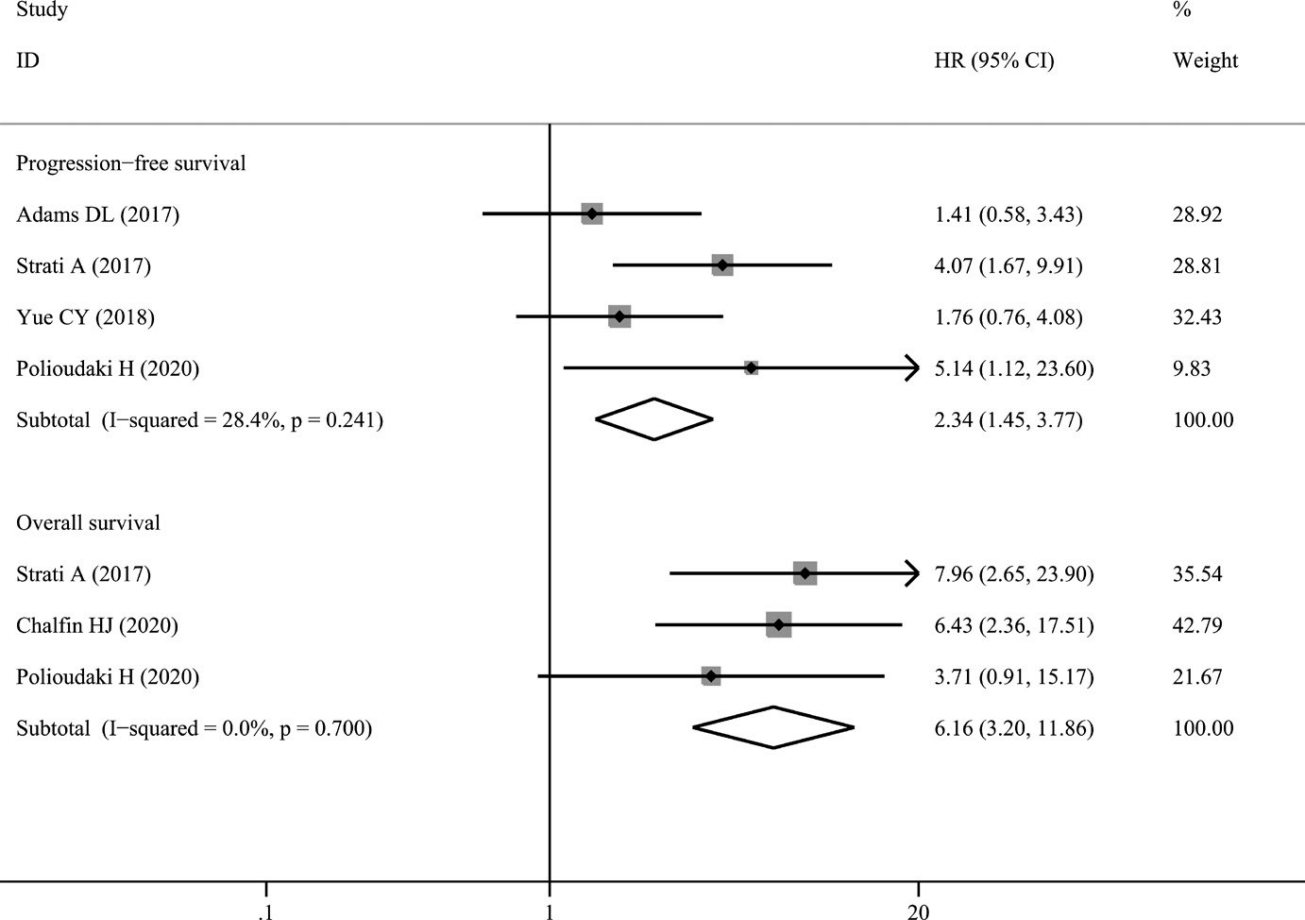
Forest plots of post‐treatment PD‐L1^+^ circulating tumor cells with survival outcomes. PD‐L1, programmed cell death‐ligand 1

The associations of post‐treatment PD‐L1^+^ CTCs with PFS and OS were analyzed in 4 studies with 201 cases and 3 studies with 199 cases (Table S1), respectively. As shown in Figure 7, post‐treatment PD‐L1^+^ CTCs were significantly associated with PFS (HR = 2.34, 95% CI 1.45–3.77, *p* < 0.001) and OS (HR = 6.16, 95% CI 3.20–11.86, *p* < 0.001).

### 3.5 Publication bias

**FIGURE 8:**
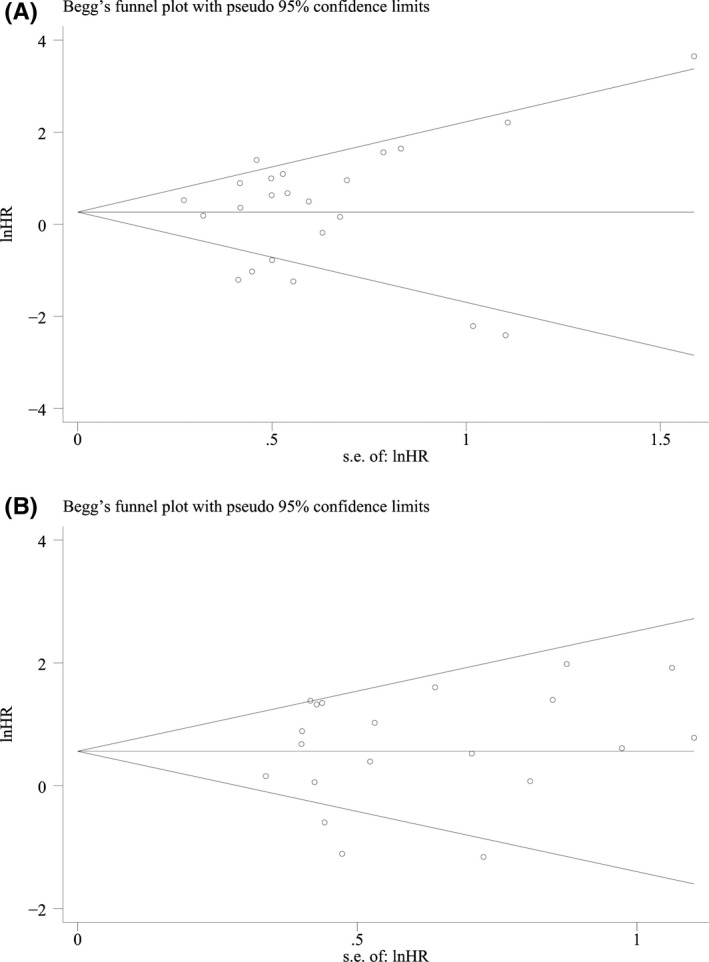
Funnel plots of pre‐treatment PD‐L1^+^ circulating tumor cells with (A) progression‐free survival and (B) overall survival. PD‐L1, programmed cell death‐ligand 1

Sensitivity analysis demonstrated that the results of our meta‐analysis were robust and not significantly influenced by any single study. The symmetric funnel plots (Figure 8) and Egger's tests (*p* > 0.05) indicated that there was no obvious publication bias.

## 4 DISCUSSION

We conducted the first meta‐analysis to evaluate the clinical application of PD‐L1 expression on CTCs in predicting the survivals of cancer patients, and to identify factors modulating the prognostic value. Overall, pre‐treatment PD‐L1^+^ CTCs may predict better survival for patients receiving ICI treatment but worse survival for patients receiving other therapies. In addition, post‐treatment PD‐L1^+^ CTCs were correlated with worse survivals in cancers.

The PD‐1/PD‐L1 axis plays a crucial role in suppressing the activation, proliferation, and promoting the apoptosis of T cells, and consequently, its upregulation on tumor tissues leads to the immune escape of tumor cells.^57^, ^58^ The PD‐1/PD‐L1 axis inhibitors disrupt the interaction between PD‐1 and PD‐L1, subsequently restore immune response toward tumor cells, and finally improve the survival outcomes of cancer patients.^4^, ^59^ Patient selection for these drugs is vital, and PD‐L1 tumor expression as a potential marker has been extensively investigated whereas there remain many unsolved issues.^3^ Some researchers have paid attention to PD‐L1 expression on CTCs. In advanced gastrointestinal tumor patients who were treated with PD‐1 inhibitors, Yue CY et al found that those with high PD‐L1 expression on CTCs had prolonged PFS and higher disease control rate compared with those with low expression.^47^ Khattak MA et al found similar results in advanced melanoma patients treated with pembrolizumab that PD‐L1^+^ CTCs predicted prolonged PFS and were more likely to be responders.^18^ In NSCLC patients receiving ICI treatment, PD‐L1^+^ CTCs were associated with better PFS and OS.^53^ However, some studies did not find a significant association between PD‐L1^+^ CTCs and survival for ICI treatment.^38^, ^48^, ^49^ Subgroup meta‐analysis by pooling these studies together showed that patients having PD‐L1^+^ CTCs and treated with PD‐1/PD‐L1 inhibitors may have prolonged PFS (HR = 0.55, 95% CI 0.28–1.08) and OS (HR = 0.61, 95% CI 0.36–1.04). Although the associations did not reach a significant level due to the small sample size (*n* = 210 for PFS and 153 for OS), PD‐L1^+^ CTCs tend to predict favorable survival prognosis for ICI treatment as more evidence are accumulating. A recent study revealed that the number of CTC detected was correlated with tumor size.^53^ Moreover, tumor size calculated in total metabolic tumor volume was significantly associated with survival and response to ICI treatment.^60^, ^61^ Thus, whether tumor size is associated with the positivity rate of PD‐L1^+^ CTCs and whether it modulates the association between PD‐L1^+^ CTCs and survival for ICI treatment need further investigation. Nonetheless, pre‐treatment PD‐L1 expression on CTCs is a potential prognostic marker for ICI treatment, which needs to be validated by more large‐scale studies in the future. In contrast to ICI treatment, our meta‐analysis showed additional evidence of a significant association between PD‐L1^+^ CTCs and survival in patients receiving non‐ICIs therapy that PD‐L1^+^ CTCs predicted significantly shorter PFS and OS.

Apart from the baseline expression, the dynamic expression of PD‐L1 on CTCs showed potentials in predicting response to anti‐tumor therapies. Several researches have found decreased number or proportion of PD‐L1^+^ CTCs upon treatment in responders but increased or unchanged expression in non‐responders.^18^, ^32^, ^47^ These results were consistent with the findings that post‐treatment PD‐L1^+^ CTCs were associated with inferior PFS and OS. Therefore, the monitoring of PD‐L1 expression on longitudinal CTC samples may help distinguish responders from non‐responders and adjust treatment strategies.

Epithelial‐mesenchymal transition is considered a pivotal process enabling tumor cells to metastasize, and vimentin is a mesenchymal marker upregulated during EMT.^54^ CTCs may also undergo EMT, and CTCs overexpressing cell surface vimentin (CSV^+^ CTCs) indicates more progressive disease.^62^ Meta‐analysis demonstrated that PD‐L1^+^ CSV^+^ CTCs were markedly associated with survival outcomes and yielded larger HRs than PD‐L1^+^ CTCs with unspecified CSV expression. The combination of these two markers may be potentially used to predict the prognosis of cancer patients.

It should be noted that the prognostic value of PD‐L1^+^ CTCs is largely modulated by the comparison models, which has not to be reported by the studies included in our meta‐analysis and should raise attention. PD‐L1 expression was not associated with survival outcomes among patients with detectable CTCs, whereas patients with PD‐L1^+^ CTCs, in comparison with those without PD‐L1^+^ CTC, had prolonged PFS and OS in overall and subgroup analyses, implying that CTCs but not PD‐L1 may underlie the association of PD‐L1^+^ CTCs with survival. However, further analyses showed no significant correlation between the effect sizes of both markers and even slightly larger effect sizes of PD‐L1^+^ CTCs than those of CTCs, indicating an independent prognostic role of PD‐L1^+^ CTCs. Nevertheless, the exact role of PD‐L1^+^ CTCs modulating response to anti‐tumor treatment and survivals needs more investigation.

Despite research progress on the clinical relevance of PD‐L1‐expressing CTCs, some issues are needing to be solved. There is no consensus on CTC enrichment and PD‐L1 detection, yet. CellSearch is the only Food and Drug Administration (FDA) approved platform of CTC enrichment and enriches CTCs by epithelial‐related markers. But some studies enriched CTCs by size‐based platforms or detected CTCs without enrichment. Two studies have detected PD‐L1 expression on CTCs enriched by a size‐based ISET platform and on matched tumor tissues, and found high concordant PD‐L1 classification.^30^, ^40^ The CTC enumeration by Epic platform which detected CTCs without enrichment process was highly consistent with that by the FDA‐approved CellSearch system.^63^ These results indicate that these non‐marker‐based platforms are also comparable and feasible for CTC enrichment. Most of the studies detected PD‐L1 by IF with different antibodies, and only a few detected mRNA expression but the cut‐offs for positive expression differed obviously.^19^, ^26^, ^35^, ^43^, ^52^ Thus, the establishment of standard procedures of CTCs enrichment and PD‐L1 detection is in urgent need.

There are some limitations to our study. First, most eligible studies have very small sample sizes. Second, there is obvious heterogeneity in the overall analysis, which may be caused by cancer types, treatments, CTC enrichment and PD‐L1 detection methods, cut‐offs, and specifically the comparison models. Third, only a few studies were performed in patients undergoing PD‐1/PD‐L1 blockade therapy. More large‐scale studies with patients of various cancers and receiving anti‐PD‐1/PD‐L1 therapy are needed in the future to validate the findings of our meta‐analysis.

## 5 CONCLUSIONS

In summary, PD‐L1^+^ CTCs are associated with better survival prognosis for ICI treatment but poor survival for non‐ICI treatment. Thus, PD‐L1 expression on CTCs may be potentially used to guide the clinical utility of ICIs in cancer patients, which needs validation in large‐scale studies in the future.

## CONFLICT OF INTEREST

The authors have no conflict of interest.

## Supporting information

Table S1

## Data Availability

The data that support the findings of this study are available on request from the corresponding author.
