# Supplementary material for: Prognostic significance of programmed cell death‐ligand 1 expression on circulating tumor cells in various cancers: A systematic review and meta‐analysis
Source: Cancer Med. 2021 Aug 23;10(20):7021–39. doi: 10.1002/cam4.4236 (PMC8525108; doi:10.1002/cam4.4236)
Supplement: Supplementary file 1 — Table S1 [file CAM4-10-7021-s001.docx]

Supplementary table 1 Association between post-treatment PD-L1^+^ CTCs and survival prognosis.

| Survival | No. of studies | HR | 95%CI | I^2^ (%) |
| --- | --- | --- | --- | --- |
|  |  |  |  |  |
| PFS | 4 | 2.34 | 1.45-3.77 | 28.4 |
| NSCLC | 1 | 1.41 | 0.58-3.43 | - |
| HNSCC | 1 | 4.07 | 1.67-9.91 | - |
| Gastrointestinal cancer^†^ | 1 | 1.76 | 0.76-4.08 | - |
| Breast cancer | 1 | 5.14 | 1.12-23.60 | - |
| OS | 3 | 6.16 | 3.20-11.86 | 0 |
| HNSCC | 1 | 7.96 | 2.65-23.90 | - |
| Genitourinary cancer | 1 | 6.43 | 2.36-17.51 | - |
| Breast cancer | 1 | 3.71 | 0.91-15.17 | - |

^†^ Treated with immune checkpoint inhibitors; PD-L1: programmed cell death ligand 1; CTCs: circulating tumor cells; PFS: progression-free survival; OS: overall survival; HR: hazard ratio. 95%CI: 95% confidence interval; NSCLC: non-small cell lung cancer; HNSCC: head and neck squamous cell carcinoma
